# Supplementary material for: Structural coordinates: A novel approach to predict protein backbone conformation
Source: PLoS One. 2021 May 20;16(5):e0239793. doi: 10.1371/journal.pone.0239793 (PMC8136669; doi:10.1371/journal.pone.0239793)
Supplement: S1 File — (PDF) [file pone.0239793.s001.pdf]

Supplementary

S1 Predictor generation and examples

One of the two main sources for feature generation is the statistics of occurrence of a certain protein sequence in the sequences of other proteins with resolved structures. Here we combine it with the RMSD distance to the sixteen protein blocks.

A predictor based on the t-statistics. For instance, let us consider one of the sixteen protein blocks $PB_j, j \in \{1, 2, 3, \ldots, 16\}$, and a 5-residue sequence *seq*. Also, let $N_{occ}(seq)$ be the number of times sequence *seq* occurs among the sequences with known structures (the training sample), $\bar{\mu}_j = \bar{\mu}_j(seq)$ be the mean distance between the structures with that sequence and the PB_j. Further, let $\bar{\mu}_j$ be the average distance between PB_j and all 5-residue fragments in the training sample, and s_j^2 be its sampling variance and N to be the size of the training sample. Then, one example of the predictors is:

$$t_j(seq) = \frac{\mu_j - \bar{\mu}_j(seq)}{s_j(seq)},$$

where

$$s(seq) = \frac{\sigma_j^2(seq)}{N_{occ}(seq)} + \frac{s_j^2}{N}.$$

Note that when $N \gg N_{occ}(seq) > 1$, $s \cong \frac{\sigma_j^2(seq)}{N_{occ}(seq)}$, the following holds:

$$t_j(seq) \cong \frac{\mu_j - \bar{\mu}_j(seq)}{\sigma_j(seq)} \sqrt{N_{occ}(seq)}.$$

Thus, the number of occurrences of as certain sequence in the sample, $N_{occ}(seq)$ is crucial for correct estimation of $t_j(seq)$. Namely, small values of $N_{occ}(seq)$ may yield unreliable estimates of $\sigma_j(seq)$ as well as $t_j(seq)$. To alleviate this, we tried various reduced alphabets.
A reduced alphabet is defined by the identity classes of the amino acids. For instance, in the following reduced alphabet example (tab.S5) for a seven-residue protein sequence all amino acids are different in positions -3, -2, 1 and 2. In positions -1, 0 and 1 amino acids $\{A, L, M, C\}$ are indistinguishable and make one identity class, amino acids $\{G, P\}$ make another identity class, $\{V, I, F, Y, W, K, R, H, D, E, N, Q, S, T\}$ make the third identity class, $\{O\}$ makes the fourth and $\{X\}$ makes the fifth:

S2 Model fitting

Here we fit a linear regression model to estimate the similarity $D_i(V)$ (1) between a protein fragment V and each of the protein blocks PB_i. Thus, each protein fragment V (we mostly use 5-residue long fragments) corresponds to 16

Table S5. An example of reduced alphabets for a 7-residue sequence fragment

-3	A V L I P M C F Y W K R H D E N Q S T G O X
-2	A V L I P M C F Y W K R H D E N Q S T G O X
-1	ALMC VIFYWKRHDENQST GP O X
0	ALMC VIFYWKRHDENQST GP O X
1	ALMC VIFYWKRHDENQST GP O X
2	A V L I P M C F Y W K R H D E N Q S T G O X
3	A V L I P M C F Y W K R H D E N Q S T G O X

values $\{D_i(V)\}_{i=1,\ldots,16}$. The fit is performed independently for each of the 16 components $D_i(V)$.
Assume $\hat{y}$ is the estimate of $D_i(V)$ for some fixed $i = 1, \ldots, 16$, and $\{x_1, \ldots, x_p\}$ are all the predictor values generated as described before. Then the model takes the following form:

$$\hat{y} = \beta_0 + \beta_1 x_1 + \cdots + \beta_p x_p, \quad (5)$$

where $\beta_0, \ldots, \beta_p$ are the regression coefficients. Commonly, the regression coefficients are estimated to minimise the squared error loss function. However, here p is large and many predictors will not be meaningful. Additionally, a number of predictors among $\{x_1, \ldots, x_p\}$ will be correlated due to the generation procedure. Hence, we first use stepwise forward-backward selection procedure to screen for the "good" predictors that show fairly high predictive capability (in terms of F-statistics), and then fit the regression model for this decreased set of predictors via least squares. The threshold for the F-statistics is determined via leave-one-out cross-validation on the training subset of the data.

S3 Tables and Figures

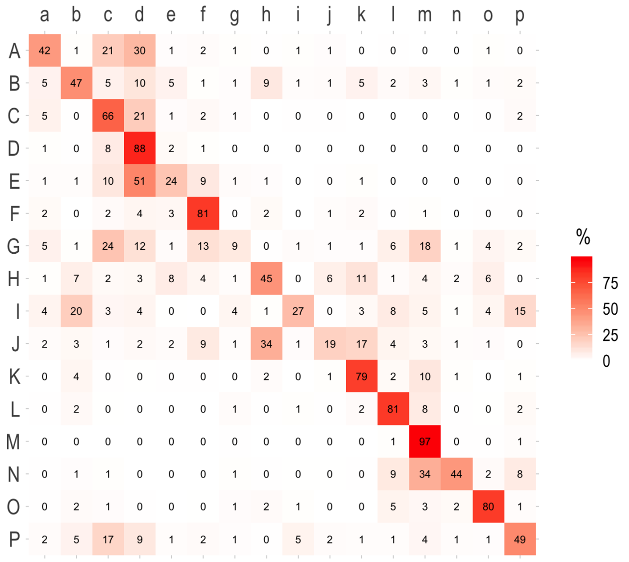

Fig S5. Cluster assignment discrepancy. Percentage of structural fragments in the learning sample shared between RMSD- and RMSDA- based clustering of those fragments.

Table S6. Cluster assignment discrepancy: RMSD vs. RMSDA

	a	b	c	d	e	f	g	h	i	j	k	l	m	n	o	p
A	42.4	0.7	20.7	29.7	0.8	1.6	0.7	0.3	0.7	0.9	0.1	0.5	0.1	0.1	0.6	0.3
B	4.9	47.2	5.4	10.5	4.9	0.7	0.9	9.1	1.2	1.4	4.9	1.9	3.3	0.7	1.4	1.7
C	5.3	0.5	65.7	20.7	1.3	2.4	0.9	0.1	0.3	0.3	0.1	0.1	0.1	0.1	0.3	1.8
D	0.6	0.4	8	87.6	2.3	0.7	0	0	0.1	0.1	0.1	0	0	0	0	0.1
E	1	1.2	9.8	51	23.8	9.1	1.4	0.9	0.1	0.4	0.7	0	0	0.1	0.1	0.3
F	2.3	0.1	1.9	3.6	3.3	81.1	0.5	2.3	0	1.1	1.9	0.5	0.7	0	0.3	0.4
G	5.4	0.7	24.4	12.3	1.4	12.6	8.8	0.3	1.4	0.9	0.9	6.2	18.2	0.6	3.9	2.1
H	0.6	6.6	2.1	2.8	7.5	3.7	1.1	44.9	0.1	6.2	11.2	1.4	3.8	1.5	6.1	0.4
I	4.4	20	3.4	4.1	0.2	0.1	3.7	0.7	27.4	0.5	2.6	8.4	4.7	0.6	3.8	15.3
J	1.6	2.8	1.3	2.4	1.5	8.7	1.1	33.6	1.3	18.7	17.1	4.3	2.8	1.1	1.3	0.4
K	0.1	3.6	0.3	0.2	0.4	0.4	0.1	1.6	0.1	0.6	79.4	2.3	9.6	0.6	0.1	0.7
L	0.5	2.4	0.2	0	0	0	1.4	0.5	0.6	0.5	1.9	81.1	8.1	0.5	0.2	2.3
M	0	0	0	0	0	0	0.3	0	0	0	0	0.6	97.4	0.3	0.3	1
N	0.4	0.8	0.6	0.1	0	0.1	0.6	0.4	0.2	0.2	0.5	8.8	33.6	43.5	2	8.1
O	0.5	1.9	1.3	0.5	0.1	0.2	1.3	2	0.6	0.1	0.1	4.8	2.9	2.2	80.4	1
P	1.5	4.9	16.9	9.2	1	1.9	0.8	0.3	4.7	1.5	1.2	1.1	3.5	1.2	0.9	49.3

The rows correspond to the RMSD-based clusters, and the columns correspond to the RMSDA-based clusters. As before, the clusters are formed out of the fragments nearest to the corresponding protein block, in terms of RMSD (capital letters) or RMSDA (small letters). The values in the represent the percentage of fragments shared by a pair of clusters: For instance, clusters 'm' and 'M' share 97.4% of fragments.

Table S7. Distances between cluster centres

	a	b	c	d	e	f	g	h	i	j	k	l	m	n	o	p
a	0	1.58	1.28	1.56	1.51	1.44	1.03	2.16	1.85	2.23	2.19	2.23	2.37	2.5	2.25	1.41
b	1.58	0	1.77	1.88	1.57	1.54	1.7	1.53	2.02	2.05	1.57	2.02	2.07	2.12	2.52	1.37
c	1.28	1.77	0	0.65	0.96	1.15	1.52	2.65	2.71	2.93	2.63	2.79	2.81	3.03	3.13	1.73
d	1.56	1.88	0.65	0	0.81	1.14	1.87	2.79	3.02	3.17	2.84	3.07	3.12	3.34	3.49	2.02
e	1.51	1.57	0.96	0.81	0	0.61	1.63	2.37	2.8	2.8	2.5	2.87	2.88	2.98	3.17	2.02
f	1.44	1.54	1.15	1.14	0.61	0	1.61	2.08	2.59	2.47	2.13	2.63	2.6	2.72	2.9	1.92
g	1.03	1.7	1.52	1.87	1.63	1.61	0	1.99	1.8	2	2.17	2.19	2.22	2.34	1.91	1.53
h	2.16	1.53	2.65	2.79	2.37	2.08	1.99	0	2.1	0.91	0.9	2.27	2.21	2.12	1.94	1.86
i	1.85	2.02	2.71	3.02	2.8	2.59	1.8	2.1	0	1.77	1.85	1.59	1.76	1.66	1.47	1.52
j	2.23	2.05	2.93	3.17	2.8	2.47	2	0.91	1.77	0	1.1	2.3	2.27	2.12	1.49	1.95
k	2.19	1.57	2.63	2.84	2.5	2.13	2.17	0.9	1.85	1.1	0	1.83	1.76	1.75	2.02	1.72
l	2.23	2.02	2.79	3.07	2.87	2.63	2.19	2.27	1.59	2.3	1.83	0	0.57	0.89	2.01	2.2
m	2.37	2.07	2.81	3.12	2.88	2.6	2.22	2.21	1.76	2.27	1.76	0.57	0	0.67	2.01	2.32
n	2.5	2.12	3.03	3.34	2.98	2.72	2.34	2.12	1.66	2.12	1.75	0.89	0.67	0	1.81	2.36
o	2.25	2.52	3.13	3.49	3.17	2.9	1.91	1.94	1.47	1.49	2.02	2.01	2.01	1.81	0	2.26
p	1.41	1.37	1.73	2.02	2.02	1.92	1.53	1.86	1.52	1.95	1.72	2.2	2.32	2.36	2.26	0

Root mean square distance (RMSD) between protein blocks, in angstroms. Protein blocks represent cluster centres and do not differ between RMSD- and RMSDA- based clusters.

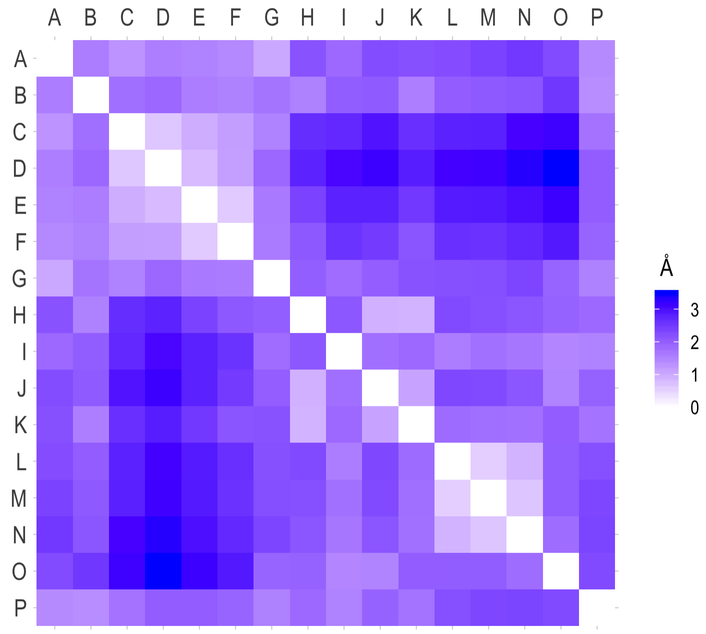

Fig S6. Heatmap of the distances between RMSD-based cluster centres.

Table S8. Prediction accuracy: Benchmarking

PB	Our Model				PB-kPred			LOCUSTRA
	PB frequency	accuracy	specificity	MCC	accuracy	specificity	MCC	accuracy
A	5.3	60.8	97.9	0.599	**67.20**	98.15	**0.69**	58.16
B	5.7	54.4	98.1	0.569	52.15	97.72	0.56	26.14
C	4.5	43.1	97.8	0.439	**58.53**	95.95	**0.58**	44.81
D	12.8	79.9	94.6	0.712	67.00	94.12	0.63	71.58
E	3.8	42.5	98.3	0.448	57.45	98.96	**0.62**	44.74
F	5.8	55.0	98.5	0.601	60.30	97.21	0.61	41.45
G	6.0	43.9	98.8	0.540	43.45	99.19	0.51	26.84
H	1.3	41.2	99.7	0.514	**61.05**	98.82	**0.64**	38.45
I	3.8	44.8	99.4	0.578	59.17	99.18	**0.63**	36,87
J	1.7	45.9	99.7	0.590	49.98	99.40	0.56	48.19
K	5.3	64.6	98.5	0.661	63.98	97.67	0.65	48.46
L	4.6	32.1	98.8	0.405	59.99	97.69	**0.62**	42.71
M	30.3	96.5	82.0	0.726	75.89	91.03	0.67	83.76
N	3.4	34.2	99.4	0.474	**62.15**	99.05	**0.65**	52.08
O	2.2	64.5	99.5	0.695	63.19	98.70	0.66	55.1
P	3.4	58.2	98.9	0.602	59.24	98.25	0.62	40.8

We assessed the prediction accuracy of our method via leave-one-out cross-validation, meaning that a complete protein and all its 5-mer fragments were removed from the training step and predicted in the validation step. The prediction accuracy for our method was assessed using the dataset *PDB30*, which is identical to the one used by PB-kPred. The prediction accuracy of PB-kPred (on *PDB30* dataset) and LOCUSTRA (a different dataset) are those reported in the original articles. [28] PB frequency stand for the frequency of the fragments from the corresponding cluster in the sample, MCC stands for the Matthews correlation coefficient. [29]

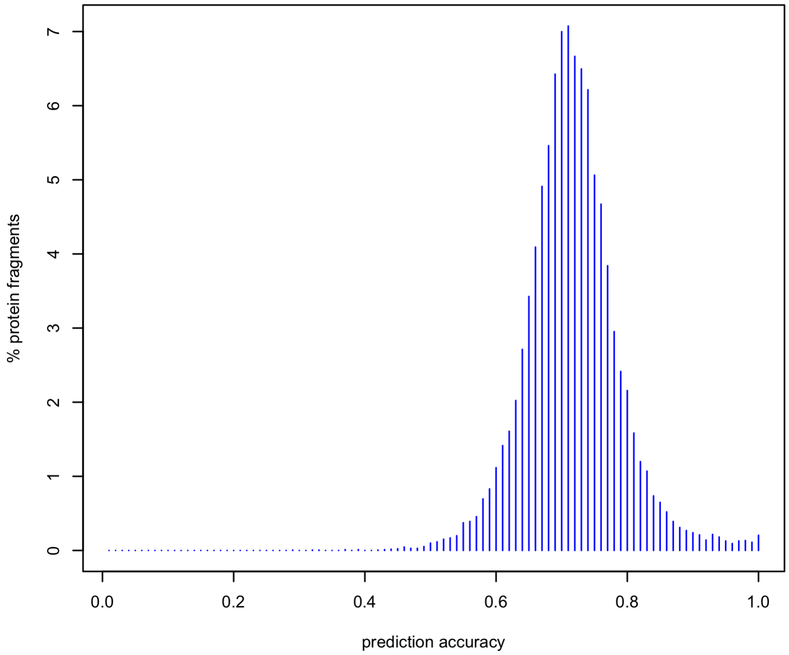

Fig S7. Distribution of the prediction accuracy calculated via cross-validation.

Table S9. Dihedral angles of the protein blocks (de Brevern et al.)

PB	ψ_1	ω_1	ϕ_2	ψ_2	ω_2	ϕ_3	ψ_3	ω_3	ϕ_4	ψ_4	ω_4	ϕ_5
A	41.139	180	75.529	13.919	180	-99.799	131.879	180	-96.269	122.079	180	-99.679
B	108.239	180	-90.119	119.539	180	-92.209	-18.059	180	-128.929	147.039	180	-99.899
C	-11.609	180	-105.659	94.809	180	-106.089	133.559	180	-106.929	135.969	180	-100.629
D	141.979	180	-112.789	132.199	180	-114.789	140.109	180	-111.049	139.539	180	-103.159
E	133.249	180	-112.369	137.639	180	-108.129	132.999	180	-87.299	120.539	180	77.399
F	116.399	180	-105.529	129.319	180	-96.679	140.719	180	-74.189	-26.649	180	-94.509
G	0.399	180	-81.829	4.909	180	-100.589	85.499	180	-71.649	130.779	180	84.979
H	119.139	180	-102.579	130.829	180	-67.909	121.549	180	76.249	-2.949	180	-90.879
I	130.679	180	-56.919	119.259	180	77.849	10.420	180	-99.429	141.399	180	-98.009
G	114.319	180	-121.469	118.139	180	82.879	-150.049	180	-83.809	23.349	180	-85.819
K	117.159	180	-95.409	140.399	180	-59.349	-29.229	180	-72.389	-25.079	180	-76.159
L	139.199	180	-55.959	-32.699	180	-68.509	-26.089	180	-74.439	-22.599	180	-71.739
M	-39.619	180	-64.729	-39.519	180	-65.539	-38.879	180	-66.889	-37.759	180	-70.189
N	-35.339	180	-65.029	-38.119	180	-66.339	-29.509	180	-89.099	-2.910	180	77.899
O	-45.289	180	-67.439	-27.719	180	-87.269	5.129	180	77.489	30.709	180	-93.229
P	-27.089	180	-86.139	0.299	180	59.849	21.509	180	-96.299	132.669	180	-92.909

Dihedral angles of protein blocks backbone used for classification and protein local structure prediction. These PB were originally designed by de Brevern et al. and used in their implementation of the PB-kPred. [12] Protein Blocks reference angles were taken from A. G. de Brevern, C. Etchebest and S. Hazout. "Bayesian probabilistic approach for predicting backbone structures in terms of protein blocks", Proteins, 41: 271-288 (2000)
